# Laryngeal Manifestations of Rheumatoid Arthritis

**DOI:** 10.1155/2013/103081

**Published:** 2013-06-25

**Authors:** A. L. Hamdan, D. Sarieddine

**Affiliations:** Department of Otolaryngology-Head & Neck Surgery, American University of Beirut Medical Center, P.O. Box 11-0236, Beirut, Lebanon

## Abstract

Rheumatoid arthritis is a destructive autoimmune disease that affects 3% of the adult population. It is characterized by the formation of both articular and extra-articular lesions with predilection for small joints. There are ubiquitous reports on the head and neck manifestations of RA with emphasis on the larynx. The laryngeal presenting features of this systemic disease may mimic a plethora of medical conditions, inflammatory and neoplastic. The main phonatory and respiratory symptoms are often subtle and misleading. This paper represents a literature review of the laryngeal manifestations of RA with emphasis on the clinical symptoms, laryngeal findings, diagnosis, and treatment. An early diagnosis of laryngeal involvement may prevent drastic complications.

## 1. Introduction

Rheumatoid arthritis is a common autoimmune disease that affects 3% of the adult population and up to 35 per 100,000 of the pediatric population [1, 2]. It is a destructive systemic disease that affects all joints in the body. The course of the disease is characterized by remissions and exacerbations, with chronicity often leading to disability. It is characterized by the formation of both articular and extra-articular lesions with predilection for small joints [3, 4]. Pressure areas are affected the most, with inflammation of the synovial membrane often leading to bony destruction and joint deformities. Extra-articular nodules may also be present in various organs of the body in up to 20% of the cases [3–7]. 

The head and neck manifestations of RA may be the sole presenting feature of this systemic disease mimicking a plethora of medical conditions, inflammatory and neoplastic. The otolaryngologic signs and symptoms of RA are protean and ill defined, with joint involvement being the most significant. These include the temporomandibular joint, the cricoarytenoid joint, and the cricothyroid joint. 

## 2. Method

A search of the literature via MEDLINE (PubMed) using rheumatoid arthritis and larynx as key words was performed by the authors. Relevant articles were reviewed, and information was retrieved and stratified based on epidemiology, clinical symptoms, laryngeal findings, diagnosis, and treatment.

### 2.1. Epidemiology

Laryngeal involvement in patients with RA is invariably underdiagnosed early in the course of the disease in view of the subtle clinical findings. A high index of suspicion is often needed to recognize laryngeal involvement especially in the presence of confounding factors such as laryngopharyngeal reflux disease and allergy. The prevalence of the laryngeal manifestations of RA has been on the rise. In a report by Lawry et al. in 1960, the prevalence of laryngeal symptoms was up to 31% [7]. Towards the end of the century, the prevalence increased to seventy-five percent. This increase can be attributed either to the enhanced awareness regarding the laryngeal involvement with RA or to the improved yield in the diagnosis. It is important to note that the clinical prevalence falls below the postmortem histopathological diagnosis of laryngeal involvement which is estimated to be up to 90% of the cases. When present, the laryngeal manifestations span an array of findings ranging from cricoarytenoid joint fixation and neuropathy of the recurrent laryngeal nerve, to myositis and presence of laryngeal nodules [8–17].

### 2.2. Laryngeal Symptoms

Autoimmune diseases in general and rheumatoid arthritis in particular can cause dysphonia or change in voice quality secondary to either functional or anatomical laryngological alterations, both of which have an impact and restrictions on quality of life. When present, dysphonia should alert general practitioners, rheumatologists, and otolaryngologists to the possible laryngeal immersion. Grossman et al. found that half patients with RA had laryngeal symptoms [18]. Similarly, several studies have reported that up to fifty percent of patients are having laryngeal involvement as the sole manifestation of this disease [19, 20]. The clinical presentation may vary from being asymptomatic to a constellation of upper aerodigestive symptoms. The array of symptoms include odynophagia, foreign body sensation, dysphagia, sore throat, lump sensation in the throat, change in voice quality, referred otalgia, and respiratory symptoms [21]. In rare cases, patients with RA may also present with symptoms of croup [22]. The change in voice quality in patients with RA may vary from mild roughness to complete—aphonia. Based on GRBAS scale where G stands for grading, R for roughness, B for breathiness, A for asthenia, and S for straining, 35% of patients with RA have grades 2 and 3 [23]. In a study by Amernik on 77 patients with recognized RA with average disease duration of 9.4 years, the most frequent complaints were foreign body sensation in 51%, hoarseness in 47%, and voice weakness in 29% of the cases. In acute phases, patients may complain of burning, foreign body sensation in the throat, and difficulty in swallowing. In chronic cases the cricoarytenoid joint (CAJ) is usually affected with resultant fixation, and airway symptoms may arise often necessitating an emergency tracheotomy [24].

Bayar et al. have reported that 66% of laryngeal symptoms when present are often attributed to CAJ involvement [16]. On the other hand, an investigation by Bienenstock et al. on 64 patients with RA showed that none had symptoms of cricoarytenoid joint arthritis [4]. Irrespective whether the involvement of the joint is acute or chronic, unilateral or bilateral, the position of the vocal cord is an important determinant of both voice quality and respiration. In cases of mild joint involvement of the joint, the mobility of the vocal cord may not be impaired and hence both phonation and breathing are unaffected. When the inflammation is moderate and one joint is involved, patients may have no or minimal airway symptoms, with occasional or mildly persistent respiratory discomfort, shortness of breath, and decrease in exercise tolerance. In cases of bilateral involvement of the joints, the clinical presentation will depend on the position of the vocal cords. If both vocal cords are immobile and cannot assume the phonatory position, that is, near total adduction, patients will present with breathiness, vocal fatigue, inability to sustain phonation, and at times aphonia. In a report by Kumai et al., aphonia secondary to hampered adduction of the vocal folds may be the presenting symptom in patients with RA necessitating arytenoid adduction [25]. On the other hand, if the vocal cords are fixed in the midline, the arthritis may endanger the patient with dyspnea and chocking. The respiratory symptoms in similar cases have been reported as early as 1880 with closer attention being paid towards the late 1950s and 1960s [13, 21]. Even in cases of acute inflammation or chronic involvement of the CAJ, patients may present with stridor that is life threatening.

The differential diagnoses of stridor in patients with RA include asthma that is refractory to medical treatment, fictitious asthma, paradoxical vocal fold movement, fixation of the vocal cords secondary to other autoimmune diseases, vocal fold paralysis secondary to recurrent laryngeal nerve injury, or the presence of a laryngeal mass. In cases of fixation of both vocal cords, the use of pulmonary function testing invariable shows evidence of extrathoracic obstruction. The characteristic changes include a forced expiratory flow at 50% lung volume/forced inspiratory flow at 50% lung volume greater than one, or forced expiratory volume in 1 second/peak expiratory flow rate greater than 10 mL/minute [26]. Electromyography is a useful test to differentiate between CAJ fixation and paralysis secondary to recurrent laryngeal nerve injury. High-resolution computerized tomography is also helpful for early detection of CAJ arthritis. The most common findings are increased density of the joint, narrowing of the joint space, ankylosis, and vocal fold thickening.

The authors recommend otolaryngologic followup and periodic laryngoscopic examination especially preoperatively in case a patient with RA is scheduled for surgery. Miyanohara et al. have reported aggravation of laryngeal rheumatoid arthritis after the use of a laryngeal mask airway in a 55-year-old woman undergoing wrist arthrodesis under general anesthesia. Aggravation of her laryngeal RA resulted in stridor postoperatively that resolved on steroid treatment [27]. Patients with RA may also present a challenge to the anesthesiologist in view of their inability to extend the neck secondary to the cervical spine ankylosis. This may necessitate the insertion of percutaneous cricothyroidotomy cannula.

Another foreseen laryngeal manifestation of RA is cricothyroid joint arthritis. In cases of involvement of the cricothyroid joint, patients will complain of limited vocal range. The authors of this paper has previously reported on the structural and functional abnormalities of the cricothyroid joint in 11 patients with advanced RA. The results indicated that almost half the patients had loss of range compared to none in controls, two-thirds had mild-to-moderate vocal fatigue compared to one-fourth of the control group, and 38% had hoarseness compared to 25% in control [28].

### 2.3. Laryngeal Findings

**Figure 1:**
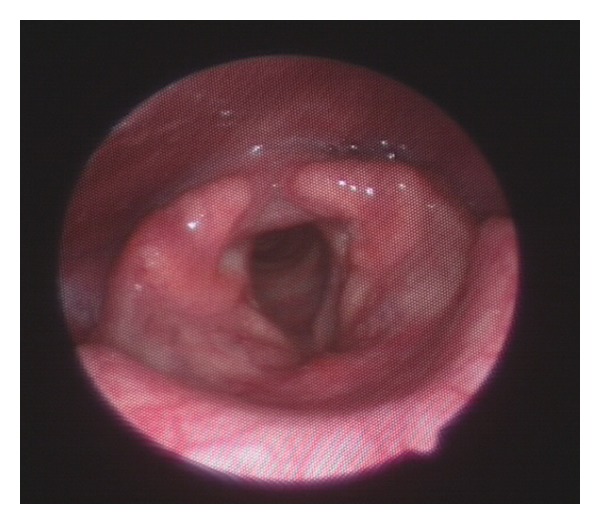
Nasopharyngeal fiberoptic endoscopic view of the larynx showing edema and deformity of both arytenoid cartilages during deep inspiration in a 37-year-old man with advanced rheumatoid arthritis.

The yield of laryngoscopic examination in patients with RA varies with the instrumentation used and the method of examination. The laryngeal manifestation varies between 13% and 75% [29–36]. The laryngoscopic findings include mucosal edema, myositis of the intrinsic laryngeal muscles, hyperemia, inflammation and swelling of the arytenoids, interarytenoid mucosa, aryepiglottic folds and epiglottis, and impaired mobility or fixation of the cricoarytenoid joint. In the early stage of the disease, the laryngeal examination may be normal. In acute involvement of the cricoarytenoid joints, signs of inflammation such as edema and redness may be present with or without impaired mobility (Figure 1). In chronic cases where ankylosis of the cricoarytenoid joint is present, one or both vocal cords may be fixed in the median, paramedian, or lateral positions.

Other laryngoscopic findings include the presence of inflammatory masses or rheumatoid nodules in the larynx and pharynx. In 1987, the American Rheumatism Association has included submucosal nodules in the laryngeal tissue in her revised criteria for the classification of rheumatoid arthritis [37]. The nodules can present as submucosal and/or subcutaneous masses in patients with autoimmune diseases. At the glottic level, these are more likely to occur in the posterior part of the vocal folds [38]. In view of the significant diagnostic dilemma in RA patients with suspicious lesions, the diagnosis can be done by excising the lesion or simply performing a fine-needle aspiration [39]. Histopathologically, these lesions carry similarities with rheumatoid nodules present elsewhere in the body. The nodule carries areas of fibrinoid necrosis surrounded by palisading epithelioid macrophages and other mononuclear cells.

Laryngoscopic findings in RA may also include the presence of Bamboo nodes. Bamboo nodes were initially described by Hosako et al. in a female patient with lupus erythematous. Endoscopic visualization shows transversally arranged cystic yellowish bamboo nodes in the submucosal space of the middle portion of the vocal folds. Similar to other laryngeal lesions in patients with RA, these nodes are more often seen in patients with active disease rather than inactive and correlates with antibody deposits [40]. These lesions are seen more commonly in females with history of phonotraumatic behavior and gastroesophageal reflux disease [41, 42]. The true incidence of these lesions is not clear despite the presence of several reports in the literature [43, 44]. In selected patients with autoimmune diseases, these laryngeal lesions have been reported in almost 80–100% of the cases [43].

### 2.4. Laryngeal Radiologic Findings

Most radiologic reports on laryngeal involvement have focused on the cricoarytenoid joint in view of its crucial role in respiration. Radiologic evidence of CAJ abnormalities in patients with RA is not commensurate with the presence or absence of laryngeal symptoms. The presence of radiologic changes may either precede or follow the clinical findings, with 58% of the cases being asymptomatic. Cricoarytenoid joint involvement can go from 25% to 72% depending on the sensitivity of the imaging technique. Jurik and Pedersen have reported evidence of osseous destruction in 45% of the cases on low-voltage radiography [45]. Using computerized tomography, the prevalence of CAJ abnormalities is higher and varies between 54% and 72%. Cricoarytenoid prominence, density and volume changes are present in almost half of the cases (46%). Other radiologic findings include subluxation in 39.9%, narrowing in the piriform sinuses in 33.3%, decrease in the CAJ space in 13.3%, and irregularities in the joint in one-fifth of the cases [16, 46]. It is worth noting that erosion of the cricoid cartilage is often mistaken for an aggressive carcinoma or tumor of the larynx. Haben has reported on a 56-year-old male with rheumatoid arthritis who presented with airway obstruction secondary to an inflammatory subglottic mass mimicking a cartilaginous neoplasm [47].

The cricothyroid joint is also a diarthrodial joint that can be affected in patients with rheumatoid arthritis. The author of this paper has previously reported on the structural cricothyroid joint abnormalities in patients with rheumatoid arthritis. Eleven patients with advanced RA underwent high-resolution computerized tomography (HRCT). The findings indicated narrowing of the CTJ in 81.8% and ankylosis in 9.1% compared with none in the control group. Almost half of the subjects had an increase in the CTJ density compared to 12.5% in the control group [28].

Table 1 summarizes the laryngeal symptoms, clinical and radiologic findings.

### 2.5. Pathophysiology of Cricoarytenoid Joint Arthritis

Impaired mobility of the vocal fold in patients with RA can be attributed to one of many possible etiologies. One is involvement of the *cricoarytenoid joint* by the rheumatoid changes. The cricoarytenoid joint is a diarthrodial joint lined by synovium and has a ligamentous capsule. The involvement of this joint may start with inflammation of the synovial lining and spreads to the articulating surfaces leading to fibrosis and later ankylosis [3, 48]. The impaired movement may be in the vertical, anteroposterior, or mediolateral directions. Histopathologic findings vary from inflammatory changes to synovial proliferation and destruction of the articular cartilage, with or without pannus [21]. The presence of these histologic changes is almost invariably based on a postmortem series by Bienenstock et al. on seven patients with RA [4]. In a study on 218 cases of bilateral fixation of the vocal folds, cricoarytenoid joint fixation was the cause in 6.3% [49].

Similarly, a report by Grossman et al. indicated the presence of CAJ involvement in only 5 out of 11 cases with RA, with less than half being symptomatic despite the joint involvement [18]. A second cause for the impaired mobility of the vocal folds is the presence of *rheumatoid nodule* in either the vocalis muscle and/or near the CAJ hindering its mobility. Erb et al. have shown the presence of these confluent nodules in conjunction with inflammation of the synovial joints, destruction of the laryngeal cartilages, and impinging on the airway [50]. A third cause for the impaired mobility is *abductor muscle paralysis.* Darke et al. have reported the presence of severe demyelination and degeneration of the recurrent laryngeal and vagus nerves together with atrophy of the laryngeal muscles with or without obliterative arteritis of the vasa vasorum [51]. A fourth possible etiology is *cervicomedullary compression* due to rheumatoid involvement of the cervical spine. Link et al. have reported this often overlooked cause of vocal fold palsy in patients with RA [52]. Watkinson has also described laryngeal amyloidosis as a rare cause of stridor in patients with RA [53].

## 3. Treatment

Early diagnosis and treatment of the laryngeal manifestation of RA are essential in preventing nonreversible sequel of this disease. The treatment may be medical, phoneatric, or surgical.

The medical treatment consists of administering steroids or nonsteroid anti-inflammatory drugs to avoid the formation of nodules and fibrosis. The effect of steroid treatment is less pronounced in cases of laryngeal nodules, probably due to the late diagnosis and the subtle clinical course of these lesions. The steroids may be given systemically or locally into the joint as reported by Habib [54]. The local injection can be administered alone or in parallel with parental treatment. A second line of treatment is the administration of methotrexate especially for the treatment of advanced cases of active arthritis. It is important to note the precipitating effect of this drug in the formation of nodulosis as a potential complication. Kerstens et al. have reported accelerated nodulosis in 5–10% of patients with RA treated with low-dose methotrexate therapy [55]. With respect to the Bamboo nodes, these lesions may be treated either surgically or conservatively. Hilgert et al. favor conservative approach to these lesions and have reported good outcome with logopedic therapy [56]. The recommendation is to start voice therapy, and if patients are still dysphonic, then steroid injection and surgical intervention are advised. The surgical treatment of these lesions consists in excision under general anesthesia using microlaryngeal suspension with preservation of the overlying mucosa.

When stridor is present, prompt recognition can be lifesaving. In cases of unilateral fixation, medialization using either injection laryngoplasty or laryngeal framework surgery is recommended. Kumai et al. have reported on arytenoid adduction for the treatment of impaired adduction of the vocal fold in a woman with RA suffering from aphonia [25]. When both vocal folds are fixed in the midline, a tracheotomy, temporary or permanent, may be indicated to alleviate the obstructed airway.

## 4. Conclusion

Rheumatoid arthritis is an autoimmune systemic disease with a wide clinical presentation. The laryngeal manifestations are often masked by the articular disability often experienced in the early and late stages of the disease. The emergence of subtle airway symptoms and or change in voice quality in patients with RA should alert the primary caring physician and specialists to the presence of laryngeal involvement. A thorough laryngoscopic evaluation is recommended to rule out cricoarytenoid joint impaired mobility. A multidisciplinary approach is needed to provide adequate laryngeal rehabilitation and alleviate the patient's suffering. Future research on cine computerized tomography with three-dimensional configurations of the arytenoid cartilages can illustrate the impact of RA as an inflammatory disease on laryngeal biomechanics and dynamic behavior of the cricoarytenoid joints during phonation and forceful breathing.

## Figures and Tables

**Table 1:** Laryngeal manifestations of rheumatoid arthritis.

Laryngotracheal symptoms	Laryngeal findings	High-resolution computerized tomographic findings
(1) Phonatory symptoms (a) Hoarseness (b) Breathiness (c) Vocal fatigue (d) Inability to project the voice (e) Complete aphonia (2) Pharyngeal symptoms (a) Dysphagia (b) Odynophagia (c) Sore throat (d) Foreign body sensation (e) Globus pharyngeus (3) Airway symptoms (a) Shortness of breath (b) Decrease exercise tolerance (c) Stridor (d) Dyspnea (e) Respiratory distress	(1) Edema (2) Hyperemia (3) Myositis (4) Impaired mobility of the vocal fold (5) Fixed vocal cords (6) Epiglottitis (7) Rheumatic nodules (8) Bamboo nodes	(1) Cricoarytenoid prominence (2) Density changes in CAJ and/or CTJ (3) Volume changes in CAJ and/or CTJ (4) Soft tissue changes in CAJ or CTJ (5) Erosion of the CAJ or CTJ (6) Ankylosis of the CAJ or CTJ

CAJ: cricoarytenoid joint. CTJ: cricothyroid joint.
